# Modeling Disorder in Pyrochlores and Other Anion-Deficient Fluorite Structural Derivative Oxides

**DOI:** 10.3389/fchem.2021.712543

**Published:** 2021-08-31

**Authors:** V. Kocevski, G. Pilania, B. P. Uberuaga

**Affiliations:** Materials Science and Technology Division, Los Alamos National Laboratory, Los Alamos, NM, United States

**Keywords:** fluorite, disorder–compounds, atomistic material modelling, pyrochlore, short range order (SRO)

## Abstract

Their very flexible chemistry gives oxide materials a richness in functionality and wide technological application. A specific group of oxides that have a structure related to fluorite but with less oxygen, termed anion-deficient fluorite structural derivatives and with pyrochlores being the most notable example, has been shown to exhibit a diversity of useful properties. For example, the possibility to undergo a transition from an ordered to disordered state allows these oxides to have high radiation tolerance. Atomistic-scale calculations in the form of molecular dynamics (MD) and density functional theory (DFT) have been extensively used to understand what drives this order/disorder transition. Here we give a brief overview of how atomistic-scale calculations are utilized in modeling disorder in pyrochlores and other anion-deficient fluorite structural derivatives. We discuss the modeling process from simple point defects to completely disordered structures, the dynamics during the disordering process, and the use of mathematical models to generate ordered solid-solution configurations. We also attempt to identify the challenges in modeling short range order and discuss future directions to more comprehensive models of the disordered structures.

## Introduction

Fluorite structural derivatives–oxides with a crystal structure related to fluorite–have attracted great interest both in fundamental research and in application. From the anion-deficient fluorite structural derivatives, pyrochlores, with a general formula A_2_B_2_O_7_ (Fd–3m space group), have attracted significant attention for various technological applications owing to their structural flexibility and special defect dynamics. They have been investigated as thermal barrier coatings (Lehmann et al., 2003; Cao et al., 2004; Schelling et al., 2004; Wu et al., 2004; Tryon et al., 2006; Winter and Clarke, 2007; Vaßen et al., 2010; Pan et al., 2012; Tanaka et al., 2017), solid oxide fuel cells (Heremans et al., 1995; Diazguillen et al., 2008; Kumar et al., 2008), solid oxide fuel cells (Wuensch, 2000; Pirzada, 2001; Yamamura, 2003; Shlyakhtina and Shcherbakova, 2012), quantum spin liquids (Anderson, 1973; Balents, 2010; Clark et al., 2014), high entropy oxides (Li et al., 2019; Zhao et al., 2019; Wright et al., 2021), and superconducting (Hanawa et al., 2001; Hanawa et al., 2002) and ferromagnetic/multiferroic (Greedan, 2006; Gardner et al., 2010; Wiebe and Hallas, 2015) materials. They have also attracted special interest as nuclear waste forms (Sickafus, 2000; Wang et al., 2000; Begg et al., 2001; Lian et al., 2003a; Lian et al., 2003b; Ewing et al., 2004; Helean et al., 2004; Lian et al., 2006; Lumpkin, 2006; Sickafus et al., 2007; Hartmann et al., 2011; Li et al., 2012; Sattonnay et al., 2013; Fischer et al., 2015; Raison and Haire, 2001; Lutique et al., 2003) and inert matrix fuel materials due to their high radiation tolerance. The high radiation tolerance of some pyrochlores is related to their ability to undergo an order-disorder transition (Subramanian et al., 1983; Lang et al., 2010; Blanchard et al., 2012; Blanchard et al., 2013; FarmerMatt et al., 2014; Popov et al., 2016; Popov et al., 2018), i.e., a low thermodynamic barrier for transition from an ordered pyrochlore to a disordered fluorite structure. In contrast, pyrochlores that have high energy cost for the order-disorder transition would amorphize under irradiation (Sickafus, 2000). Understanding the disordering process and its impact on properties is critical for a number of other applications, including ionic conductors (Kreller and Uberuaga, 2021). We note that, in contrast, spinels (AB_2_O_4_), which have a crystal structure related to rocksalt and have ordered cation vacancy sites, show opposite correlation between disordering energies and amorphization resistance compared to pyrochlores (Uberuaga et al., 2015), i.e., spinels that are more difficult to disorder exhibiting higher resistance to amorphization. Further, we use the term ‘disorder’ to mean chemical disorder in a crystalline matrix, while ‘amorphization’ refers to the loss of crystallinity.

**FIGURE 1:**
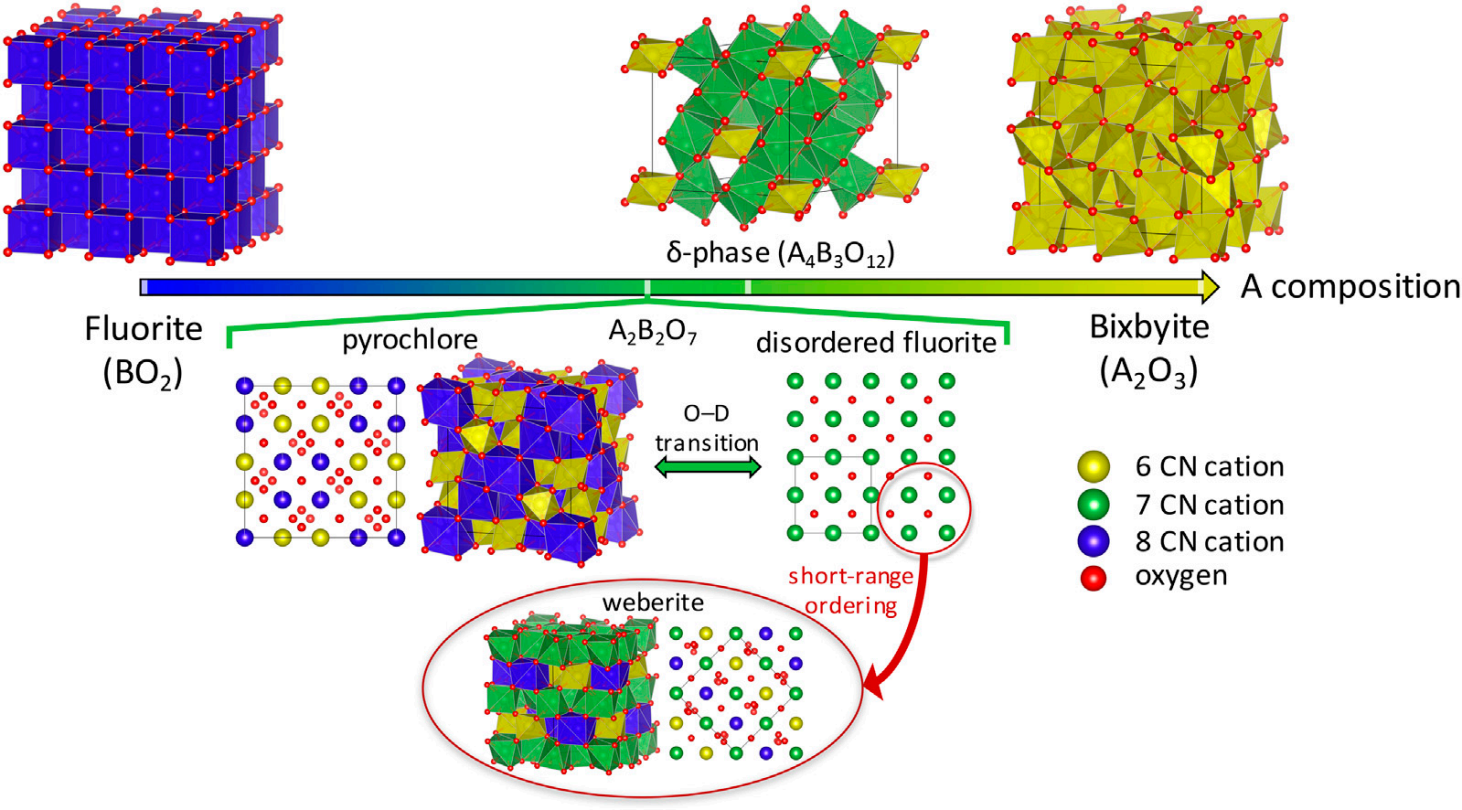
Schematic representation of the anion-deficient fluorite structural derivative oxides, going from a fluorite (BO_2_) to a bixbyite (A_2_O_3_) crystal structure. The cationic sites with coordination number (CN) of 6, 7 and 8 are shown as yellow, green, and blue spheres and polyhedra, respectively, while the oxygen atoms are shown as red spheres. The lower half highlights how, upon disordering, a fluorite-derivative structure such as pyrochlore can undergo an order-disorder transition to a structure resembling disordered fluorite on the long-range, but has short-range order more accurately described by a weberite-structure.

The pyrochlore crystal structure can be described as an anion deficient 2 × 2 × 2 superstructure of a fluorite (BO_2_), having two ordered cation sites (A in 16*d* and B in 16*c* Wykoff positions), two ordered oxygen sites (48*f* and 8*b*), and an ordered oxygen vacancy site (8*a*) (see Figure 1). It has been commonly accepted that the order-disorder transition is driven by the formation of cation antisite pairs and oxygen Frenkel pairs. Another anion deficient fluorite derivative termed as δ-phase (see Figure 1), with the general formula A_4_B_3_O_12_, is also known to exhibit a high radiation tolerance that can be attributed to the transition from an ordered δ-phase to a disordered fluorite (Sickafus et al., 2007) structure, also promoted by the formation of cation antisites and oxygen Frenkels (Sickafus et al., 2007; Stanek et al., 2009). Generally, such a transition occurs under high energy irradiation (Sickafus, 2000; Lian et al., 2003a; Lian et al., 2003b; Helean et al., 2004) (though disorder can also be induced by temperature and synthesis conditions), and thus modeling studies of the order-disorder transition have been focused on relating the formation of these defects with radiation tolerance of pyrochlores and δ-phase. A further increase in the O vacancies gives the bixbyite structure with A_2_O_3_ composition, having 1/4 O vacancies in the fluorite structure. However, unlike the pyrochlore and the δ-phase, the disordered bixbyite does not have a fluorite structure, and thus we are not going to discuss it further in this review.

**FIGURE 2:**
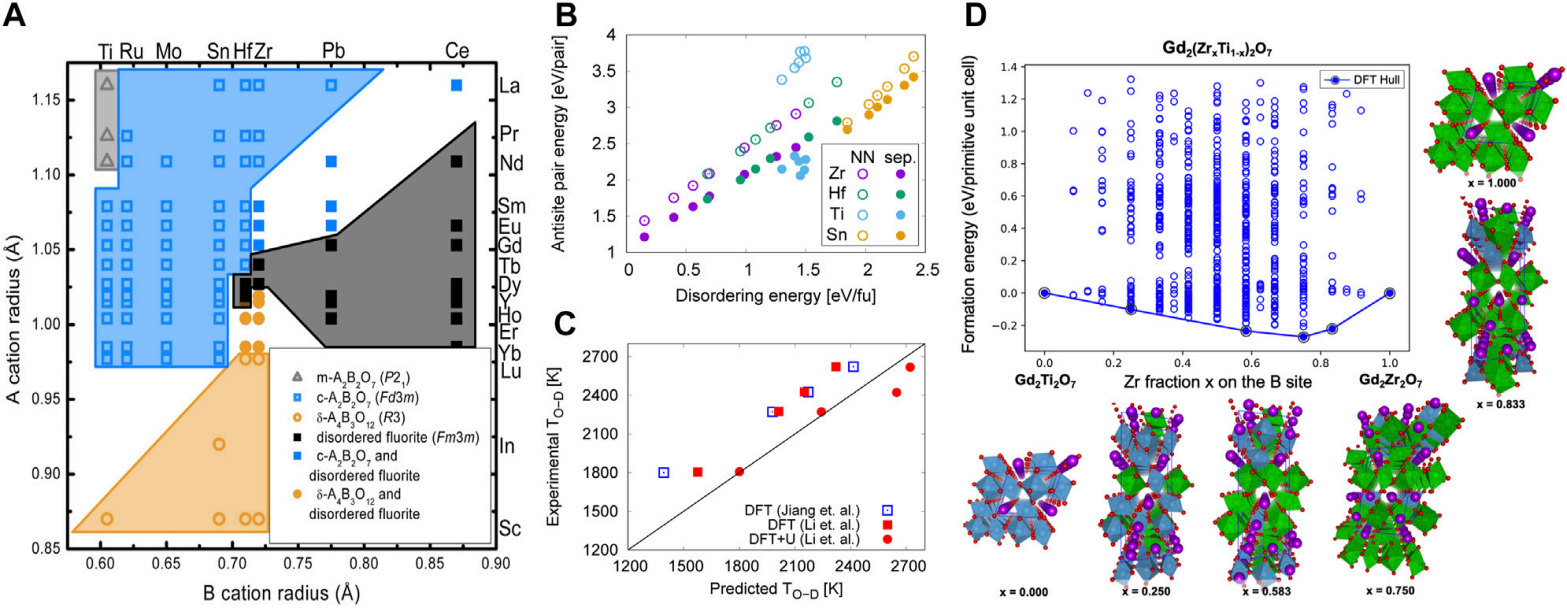
**(A)** Structure map for A_3_+B_4_+O_4_–x compounds from experimentally reported structures. Hollow gray triangles denote the monoclinic A_2_B_2_O_7_ structure, hollow blue squares denote A_2_B_2_O_7_ pyrochlore, and hollow orange circles denote the rhombohedral Γ-phase A_4_B_3_O_12_. Solid black squares denote disordered fluorite. Solid symbols of the same shape as stable ordered structures denote that both the ordered structure and disordered fluorite have been observed. Reproduced from (Stanek et al., 2009). **(B)** Formation energy of cation antisite pairs in four families of rare earth (RE) pyrochlores, RE_2_B_2_O_7_ (B = Zr, Hf, Ti, Sn), as a function of their disordering energies (calculated as described in (Jiang et al., 2009) and antisite separation distance. The formation energies when antisites are nearest neighbors (NN) and are separated (sep.) are shown with filled and open symbols, respectively. Adapted from (Uberuaga et al., 2014). **(C)** Comparison of the calculated order-disorder (O–D) transition temperature with experimental data (Stanek and Grimes, 2002) and (Rushton et al., 2004) for selected pyrochlores. The DFT results by (Jiang et al., 2009) and (Li et al., 2015) are shown in blue and red squares, respectively, and the DFT + U results by (Li et al., 2015) are shown in red circles. Reproduced from (Li et al., 2015). **(D)** Calculatedmixing energies per primitive unit cell for the 704 unique configurations enumerated within the Gd_2_(Zr_x_Ti_1_–x)_2_O_7_ chemistries. The DFT predicted convex hull is represented as a solid line. The atomistic configurations for the identified ground-state ordered structures on the convex hull are illustrated using purple and red spheres for Gd and O, respectively, while Ti and Zr polyhedra are depicted in blue and green colors, respectively. Reproduced from (Pilania et al., 2019).

Studies of disordered pyrochlores suggest that the short-range structure is more ordered than is described by the random disordered fluorite structure (Liu et al., 2004; Ushakov et al., 2007; Saradhi et al., 2012; Norberg et al., 2012; Zhang et al., 2013). Recently it was shown using neutron total scattering experiments with pair distribution function (PDF) analysis that disordered pyrochlore (Ho_2_Zr_2_O_7_) has the atomic arrangement of the defect fluorite on a long-range scale, but the short-range ordering is better explained by a partially ordered orthorhombic phase that is well described by a weberite-type (C222_1_ space group) structural model (Shamblin et al., 2016). Weberite is also a superstructure of fluorite with a unit cell of 2×√2×√2; it is more ordered than disordered fluorite but less ordered than pyrochlore. Unlike pyrochlore where A and B cations occupy the 8- and 6-fold coordinated sites, respectively, in weberite the A and B cations occupy 8- and 6-fold coordinated 4*b* sites, respectively, while the 7-fold coordinated 8*c* site is equally occupied by both cations (see Figure 1). Note that 2-2-7 chemistries with weberite structure containing rare-earth cations have not been experimentally reported (Cai and Nino, 2009) (see Figure 2A), and, as far as we have found, there is only one experimental report of a 2-2-7 chemistry, Ca_2_Sb_2_O_7_, that undergoes a pyrochlore-to-weberite transformation at 973 K (Brisse et al., 1972).

In the past three decades, atomic scale calculations, by means of molecular dynamics (MD) simulations with interatomic potentials and density functional theory (DFT) calculations, have been extensively used to understand the disordering process in pyrochlores by calculating the intrinsic defects formation energies (Wilde, 1998; Chartier et al., 2002; Minervini et al., 2002; Minervini et al., 2004; Panero et al., 2004; Rushton et al., 2007; Sickafus et al., 2007; Chen et al., 2008; Chen and Tian, 2010; Gunn et al., 2012; Chen et al., 2014; Uberuaga et al., 2014; Li et al., 2015; Li and Kowalski, 2018; Yang et al., 2019), looking at dynamical processes (Chartier et al., 2002; Chartier et al., 2005; Devanathan and Weber, 2005; Todorov et al., 2006; Rushton et al., 2007; Chartier et al., 2009; Devanathan et al., 2010; Xiao et al., 2010; Wang et al., 2013; Uberuaga et al., 2014) (Xiao et al., 2015; Dong et al., 2017), and using models of disordered configurations for DFT calculations (Wolff-Goodrich et al., 2015; Jiang et al., 2009; Solomon et al., 2016; Finkeldei et al., 2017; Pilania et al., 2019; Kowalski, 2020; Matsumoto et al., 2020). The simplest way to study the disorder in a material using atomistic scale calculations is to obtain the formation energies of intrinsic defects in ordered systems, which are generally accessible at a much lower computational cost than completely disordered systems. Although these dilute-limit defect energies only reflect the initial steps of disorder, they can give valuable information and qualitative agreement with experiments. In the case of pyrochlores, the basic premise would be that the formation energy of point defects, such as cation antisites, correlates with the energy to fully disorder the crystal (Uberuaga et al., 2014). A step between the study of intrinsic defects and complete disorder is analyzing dynamical processes in pyrochlores, such as threshold displacement energies, heavy ion track simulations, collision cascades, and defect accumulation. Such studies provide additional information on the energetic cost to disorder the system beyond the static intrinsic defect studies.

Performing similar simulations using ab initio MD (AIMD) is prohibitively expensive, thus a static model of the disordered fluorite is needed for DFT calculations. Such models of the disorder can be generated by arranging the A and B cations and the oxygen vacancies in a fluorite supercell, creating a special quasirandom structure (SQS) (Zunger et al., 1990) or a small set of ordered structures (Jiang and Uberuaga, 2016) such that their most relevant radial correlation functions mimic a perfectly random structure. These models provide the opportunity to compare the properties of both the ordered (pyrochlore) and disordered (fluorite) phases. Another approach, which can also be used to examine alternative cation arrangements and their temperature dependence via Monte Carlo, is cluster expansion (CE). Below we summarize how the above-mentioned methods are used in analyzing the order-disorder transition in anion deficient fluorite derivative phases, with a focus on pyrochlore as this system has been most extensively studied.

## Defect Formation

The defect formation energies of pyrochlores were initially studied beginning more than 20 years ago using interatomic potentials (Wilde, 1998; Chartier et al., 2002; Minervini et al., 2002; Minervini et al., 2004; Rushton et al., 2007; Sickafus et al., 2007; Gunn et al., 2012), while recently more accurate DFT calculations have also been utilized (Panero et al., 2004; Chen et al., 2008; Zhang et al., 2009; Chen and Tian, 2010; Chen et al., 2014; Uberuaga et al., 2014; Li et al., 2015; Li and Kowalski, 2018; Yang et al., 2019). Despite the higher defect formation energies calculated using interatomic potentials compared to DFT, both types of studies show an apparent correlation between decreased cation antisite pair formation energies and the radiation tolerance of pyrochlores. Both also report a peak in the defect formation energies at Gd in the A_2_Ti_2_O_7_ series, which agrees with the experimentally observed highest critical amorphization temperature for Gd_2_Ti_2_O_7_ (Lian et al., 2003a). (Uberuaga et al. 2014) saw precisely these relationships in Ti and other pyrochlore chemistries (Figure 2B).

Once the oxygen Frenkel is coupled with a cation antisite pair, the formation energy of such clusters becomes lower than the isolated defects (with a few exceptions (Li et al., 2015; Li and Kowalski, 2018)). This enhanced stability of the clusters indicates that cation antisites cause local oxygen disorder, and hence, drive the formation of disordered fluorite structure. (Stanek and Grimes, 2002) and (Rushton et al., 2004) correlated the formation energies of these clusters with the experimental pyrochlore-to-disordered-fluorite transition temperatures for A_2_Hf_2_O_7_ and A_2_Zr_2_O_7_, respectively, showing a clear linear relation. Studies with potentials also report a threshold cluster formation energy below which disordered fluorite is preferred, demonstrating why some chemistries do not form the pyrochlore structure. (Yang et al., 2019) evaluated the defect formation energies in off-stoichiometric A_2_B_2_O_7_ (A = La, Nd, Gd; B = Zr, Hf, Sn), demonstrating how the synthesis methods, i.e., excess of A_2_O_3_ or BO_2_, can influence the radiation tolerance of pyrochlores.

The most important observations from these studies are that the energetics of cation antisites correlate with the amorphization resistance of the material and that cation and oxygen disordered are coupled. The correlation between disordering and amorphization has been explained by (Sickafus, 2000) as related to an inherent ability of the structure to create low-energy defects that do not drastically increase the stored energy in the material. On the other hand, that cation and anion disorder are linked is a consequence of the differences in preferred coordination in the A and B cations–8 and 6, respectively. As the cations are disordered, they will naturally drive rearrangement of the oxygen to best retain their preferred coordination, and these modeling results directly demonstrate this fact. However, this behavior is not universal, as we will see in our discussion of the δ-phase structure below.

## Dynamical Processes

Threshold displacement energies (TDEs) can characterize the resistance of a given sublattice to short-range displacements in a specific crystallographic direction, by looking at the response of the crystal to a primary knock-on atom (PKA). The TDE of pyrochlores have been studied using classical MD Chartier et al., 2002; Devanathan and Weber, 2005; Dong et al., 2017) and AIMD (Xiao et al., 2010; Wang et al., 2013; Xiao et al., 2015). While both MD and AIMD give the same trend in terms of which species are easiest to displace, the calculated TDEs do not correlate with the experimental radiation tolerance of the studied A_2_Ti_2_O_7_ pyrochlores.

More detailed understanding of the main mechanisms that promote the formation of disordered fluorite, and subsequently an amorphous phase, can be gained by investigating dynamical processes during collision cascades. Collision cascades in La_2_Zr_2_O_7_ (Chartier et al., 2003) and Gd_2_B_2_O_7_ (B = Ti, Zr, Pb) (Todorov et al., 2006; Rushton et al., 2007) display very similar behavior, with significantly higher number of O defects compared to cation defects at the end of the simulation. Despite this, the oxygen-oxygen radial distribution function of the studied pyrochlores shows no hints of amorphization. A closer look into the equilibrated structures shows that most of the displaced O atoms in the studied pyrochlores recovered to an equilibrium site (48f, 8b or 8a), except for in Gd_2_Ti_2_O_7_ where the formed O defects are in interstitial sites.

Another way to study disordering processes is by simulating swift heavy ion irradiation tracks using thermal spikes. By such simulations in Gd_2_Zr_x_Ti_2-x_O_7_, (Devanathan et al., 2013) showed that an amorphous area of ∼2 nm forms in Gd_2_Ti_2_O_7_, which shrinks with increasing Zr content, and completely disappears in Gd_2_Zr_2_O_7_. The findings agree with experiments that show that Gd_2_Ti_2_O_7_ becomes amorphous under swift heavy ion irradiation while Gd_2_Zr_2_O_7_ forms disordered fluorite. A closer look into the disordering process in Gd_2_Ti_2_O_7_ showed a formation of Gd vacancies, Ti interstitials, and Gd-on-Ti antisites that facilitate amorphization. On the other hand, only Gd and Zr antisite pairs are observed in Gd_2_Zr_2_O_7_, which dissipate the excess energy.

As we discussed previously, the tendency of a pyrochlore to disorder, i.e., to form a disordered fluorite structure, is correlated with the formation energy of cation antisites and oxygen Frenkels. A more complete picture of disordering in the pyrochlore structure can be gained by introducing defects in various concentrations, and using MD to simulate the disordering process over time. Using classical MD simulations of La_2_Zr_2_O_7_ pyrochlore, (Chartier et al., 2005) showed that cation antisites induce disorder on the oxygen sublattice, indicating that the transition to a disordered fluorite is driven by cation disorder. Later (Chartier et al., 2009) extended their study to Gd_2_Zr_x_Ti_2-x_O_7_ solid solutions, showing a decrease in radiation tolerance with increasing Ti content, while (Devanathan et al., 2010) compared the two end-member compositions. Both studies show that the amorphization of pyrochlores is a two-step process: initially cation antisites and oxygen Frenkels are formed, with subsequent damage increasing the concentration of cation Frenkels, especially Ti-Ti dumbbells which resist recombination, leading to amorphization. These studies reveal that simply introducing cation disorder leads to disordering of the entire structure–the anions disorder “spontaneously” in the presence of cation disorder.

Together, these dynamical simulations both reinforce the conclusions obtained from the static calculations of defects but also reveal new details of the dynamical processes underlying the formation of disorder. During irradiation, oxygen is always easier to displace, but unless cations are also displaced, that disorder oxygen structure will not persist. Cation antisites are the driver of disorder on both sublattices. However, at some defect concentration, the lattice becomes unstable and cation Frenkels start to form. The lack of dynamical recovery leads to an exceedingly high stored energy for some chemistries, which causes amorphization. Thus, the susceptibility to amorphization is a combination of both the energetic cost of the defects that are unavoidably introduced during irradiation and their inability to heal or recombine. What is still missing in the literature is a model that can actually predict the rate of amorphization for a given material as a function of temperature and dose. We suspect this is due to the shear difficulty in developing such a model for even one compound, given all of the details that must be addressed. However, such a model for even one compound would provide important insight into the most critical factors describing the kinetics of amorphization and recovery.

## Structural Disorder

As mentioned, one can use SQS to model the disordered fluorite structure with DFT, and evaluate the energy for disordering as the energy difference between the ordered pyrochlore and the disordered SQS. (Jiang et al., 2009) used this approach to calculate the disordering energy for a set of rare-earth pyrochlores, A_2_B_2_O_7_ (A = Pr, Nd, Sm, Gd, Tb, Dy, Er; B = Ti, Zr, Hf, Sn), while (Li et al., 2015) focused on a small subset of pyrochlores (Gd_2_Zr_2_O_7_, Gd_2_Hf_2_O_7_, Sm_2_Zr_2_O_7_, Tb_2_Zr_2_O_7_). Both related the disordering energy with amorphization tendencies and estimated the order-disorder temperature, *T*_OD_, showing good agreement with experiments (Figure 2c) and highlighting the relevance of the disordered state in understanding the properties of these materials. Using the calculated *T*_OD_, (Jiang et al., 2009) were also able to explain the difficulties in synthesizing of some pyrochlores. More importantly, as highlighted in Figure 2B, these types of calculations (Uberuaga et al., 2014) reveal a direct correlation between the energetics of cation antisite pairs and full disorder in the system.

Recently, Shamlin et al. showed experimentally that the weberite-like structure, which has a random cationic distribution on the 8*c* site (7-fold coordinated site), best represents the short-range order in the disordered structure (Shamblin et al., 2016). This has inspired DFT calculations of weberite-type disordered structures (Solomon et al., 2016; Finkeldei et al., 2017; Kowalski, 2020; Matsumoto et al., 2020), which show that disordered fluorite has higher energy than disordered weberite. It is argued that this indicates a possibility of forming weberite-type short-range ordering. However, both (Kowalski, 2020) and (Matsumoto et al., 2020) also report that some A_2_B_2_O_7_ chemistries have weberite-type as a lower energy configuration, in disagreement with experiments that report either pyrochlore or δ-phase (Figure 2A). This is a curious result as no 2-2-7 chemistries containing +3 and +4 cations have been experimentally reported to have the weberite structure (Cai and Nino, 2009). On the other hand, calorimetry experiments indicate that the short-range order described by weberite is higher in energy than pyrochlore (Solomon et al., 2016; Finkeldei et al., 2017). The discrepancy between experiments and modeling can be explained for some chemistries, namely A_2_Zr_2_O_7_ and A_2_Hf_2_O_7_ (A = Ho, Er, Yb, Lu), which form δ-phase (see Figure 2A), suggesting δ-phase might be preferred and thus should be examined. (Matsumoto et al., 2020) also used the vibrational entropy of the Yb_2_Ti_2_O_7_ pyrochlore and weberite to find the weberite-to-pyrochlore ordering transition temperature, showing that the pyrochlore ordering is preferred at temperatures above 500 K. Nevertheless, the predicted ordering transition temperature is not low enough to explain why the weberite-type structure has not been experimentally observed.

Finally, there is a possibility of even greater ordering when multiple cations are introduced into one of the sublattices. Calculations of such structures can give further insight into the drivers of ordering and the relationship between cation and anion structure. (Pilania et al., 2020) have investigated precisely this possibility in Gd_2_(Ti_1-x_Zr_x_)_2_O_7_ using cluster expansion coupled with DFT calculations. As highlighted in Figure 2D, their calculations indicated that Gd_2_(Ti_0.25_Zr_0.75_)_2_O_7_, termed a double pyrochlore, has the lowest formation energy on the convex hull. The enhanced stability of this composition was attributed to a special configuration that allows for 7-fold coordinated Zr sites while maintaining the preferred 6-fold coordination of Ti-atoms. This kind of cation-induced change in the local oxygen coordination is what is behind the formation of weberite-like short-range order.

In our view, one of the most important results from these calculations is that the energetics of the individual cation antisites correlate very strongly with full disorder. This directly links the original calculations of isolated defects and the fully disordered structure. However, more recent experiments and calculations of disordered structures also reveal what we might call a conundrum. It is now abundantly clear that short-range order exists in the disordered and even amorphous materials. However, the calculations indicate that structures built on these short-range ordered motifs are actually more stable than pyrochlore. We will discuss this in greater depth below. Finally, the unexpected stability of Gd_2_(Ti_0.25_Zr_0.75_)_2_O_7_, in which Zr is able to attain its preferred 7-fold coordination, suggests that, in the pyrochlore structure, Zr is frustrated. This may explain why Zr-based pyrochlores tend to be fast ion conductors, as the oxygen struggles to maintain low-energy coordination in any one configuration.

## Other Phases

The δ-phase was initially presented as having a higher radiation tolerance as compared to pyrochlores by (Sickafus et al., 2007), which they correlate with the lower formation energy of defect clusters. Following these findings, (Stanek et al., 2009) performed a computational study of the cation ordering in A_4_B_3_O_12_ and calculated the disordering energy with respect to the disordered fluorite. (Interestingly, these compounds have not been experimentally observed to have cation order.) They then evaluated the *T*_OD_ using the same approach as (Jiang et al., 2009). The *T*_OD_ were found to be generally low, even lower compared to most of the pyrochlores from study, indicating that the δ-phase should disorder much more easily, and thus have high radiation tolerance, as initially indicated by static MD calculations (Sickafus et al., 2007).

Until now our discussion has focused on the specific compositions A_2_B_2_O_7_ and A_4_B_3_O_12_. However, it is possible to sample the whole phase space between BO_2_ and A_2_O_3_ for any stable ordered fluorite structural derivative compounds. This is what (Bogicevic et al., 2001; Bogicevic and Wolverton, 2003) and (Predith et al., 2008) did using cluster expansion to find the lowest energy ordering in the ZrO_2_–A_2_O_3_ (A = Y, Sc) and ZrO_2_–Y_2_O_3_ phase spaces, respectively. In addition to the experimentally observed δ-phases (Y_4_Zr_3_O_12_ and Sc_4_Zr_3_O_12_), both report Y_2_Zr_5_O_10_ and Sc_2_Zr_5_O_10_ to be stable with respect to cubic ZrO_2_, while (Predith et al., 2008) also report an additional Y_2_Zr_4_O_11_ stable phase. Furthermore, Predith et al. show that only Y_2_Zr_4_O_11_ and Y_4_Zr_3_O_12_ phases are stable with respect to the monoclinic, lowest energy ZrO_2_. None of these studies found the pyrochlore configuration to be stable, in agreement with experiments where only the stable δ-phase is observed for these compositions. In the reported stable structures, the oxygen vacancies align along the <111> direction and are always third nearest neighbors to each other, showing that cluster expansion can provide information on the short-range ordering in solid solutions. Such studies have been performed for other crystal structures, such as spinels (Jiang et al., 2012; Pilania et al., 2020), but have not been applied to fluorite derivatives as extensively because of the need to account for the coupling of disorder on the cation and anion sublattices.

These calculations both complement and contrast with the behavior we have described for pyrochlore. First, that the δ-phase structure is easy to disorder suggests it has high radiation tolerance, as has been observed (Sickafus et al., 2007). However, this structure also exhibits anion ordering despite the disordered cations, indicating that cation disorder does not always force anion disorder. Oxygen vacancy-vacancy repulsion, presumably, overcomes any coupling with the cation disorder. Further, as demonstrated by there can be a variety of other stable structures for some of these chemistries, suggesting that there is not one dominant phase and that short-range order in disordered materials may be related to these other structures as well. This has not been considered in prior research, to the best of our knowledge.

## Discussion and Outlook

The computational studies we have examined have provided invaluable insight into the nature of disorder in anion-deficient fluorite derivatives. It is only through atomistic modeling that the relationship between disordering and radiation tolerance has been established. However, there are a number of questions that arise from past work that can be uniquely addressed by future modeling efforts.

Classical MD simulations provide a good understanding of the processes that drive the disordering of pyrochlores to a disordered fluorite structure. However, the accuracy of MD simulations is dependent on the quality of the interatomic potential and current potentials do not capture all of the nuances of the true quantum system, such as the formation of 7-fold coordinated Zr sites predicted using DFT in Gd_2_(Ti_0.25_Zr_0.75_)_2_O_7_ (Pilania et al., 2019). Thus, new potentials could improve the understanding of the disordering processes in pyrochlores. Alternatively, AIMD using DFT forces can be performed directly to understand the disordering processes by introducing different defects in various concentrations in the simulation cells, similarly to some of the classical MD studies discussed above. Such simulations can validate classical results and provide new insight into how chemical bonding drives unique behavior. While AIMD simulations are currently computationally highly expensive, current improvements in code efficiency and computational architectures can make such simulations possible.

Using DFT calculations, SQS models of disordered fluorite provide a reasonable estimate of the *T*_OD_ of pyrochlores (Jiang et al., 2009; Li et al., 2015), with *T*_OD_ calculated with DFT + *U* being in very good agreement with experiments (Li et al., 2015). A more accurate estimate of *T*_OD_ can be obtained by accounting for vibrational entropy, as has been demonstrated in spinels (Jiang et al., 2012). Introducing the vibrational entropy to the defect formation energies can also provide more rigorous defect formation free energies, as well as a possibility to evaluate the defect concentrations at finite temperatures. A critical unknown is how equilibrium defect concentrations depend on the state of disorder–all defect calculations that have been done are for the fully ordered state and may not be representative of the disordered material.

We have discussed how cluster expansion is a powerful tool for studying the energetics of disorder, such as finding order in solid-solutions of Gd_2_(Ti_1-x_Zr_x_)_2_O_7_ (Pilania et al., 2019) and the mixing between BO_2_ and A_2_O_3_ (Predith et al., 2008). Therefore, cluster expansion can be employed to study the change from pyrochlore (ordered) to fluorite-type (disordered) structure in the studied A_2_(B_1-x_B’_x_)_2_O_7_ solid solutions, accounting for mixing on the cationic sublattice between A, B and B′ cations in addition to introducing O in the vacant 8*a* sites. While there might be challenges in developing cluster expansion models that account for the coupled order between the cation and anion sublattices, this is an integral step that must be undertaken to fully understand the nature of the short-range order in disordered pyrochlores.

Even if the disordered state is properly understood and described, this is only the beginning. To predict and ultimately design materials that leverage the disordered state, we must also know the impact on properties. As we have already noted, disordered materials have unique properties distinct from their ordered counterparts. This fact has mostly been revealed by experiments with relatively little work by modeling. There is a need, once reasonable models of the disordered state have been established, to understand the structure-property relationship of those systems. Structural properties such as short-range order and percolation networks can have dramatic effects on transport and these relationships have received scant modeling attention (Kreller and Uberuaga, 2021).

Finally, the biggest question that arises from this review is the nature of the ordering in the presumably disordered fluorite structure of these materials. Experiments indicate that short range order is present. This should be expected, as no natural material is truly random–indeed, cluster expansion studies of spinel show that short range order persists to extremely high (and unphysical) temperatures (Pilania et al., 2020). However, the random structure presents a useful conceptual limit that has been demonstrated to correlate with experimental observables such as the order-disorder transition temperature and the critical temperature for amorphization. Thus, the random structure does capture some of the physical behavior of the real materials. The key question then is when is it critical to account for the short-range order to accurately understand and predict the properties of these materials. For example, it seems clear that the nature of the short-range order is essential for understanding mass transport in these materials (Kreller and Uberuaga, 2021). However, it isn’t necessary to understand chemical trends in thermodynamic behavior. Future work must elucidate not only the nature of the short-range order, but when it must be accounted for.
